# Independent director compensation and stock price collapse: Inhibition or promotion—based on a financial background perspective

**DOI:** 10.1371/journal.pone.0289986

**Published:** 2023-08-10

**Authors:** Guoping Dong, Guifen Ma, Shanqiu Liu

**Affiliations:** Accounting Institute, Guangzhou Huashang College, Guangzhou, China; Yunnan Technology and Business University, CHINA

## Abstract

This paper takes the financial independent directors’ compensation of listed companies from 2014 to 2020 as the research object and uses empirical analysis to study whether the compensation of financial independent directors promotes or inhibits stock price collapse. The research results show that there is a significant positive correlation between the compensation of financial independent directors of listed companies and stock price collapse. In state-owned enterprises, the compensation of financial independent directors has an inhibitory effect on stock price collapse, but it is not significant. In non-state-owned enterprises, the compensation of financial independent directors has a significant promoting effect on stock price collapse. Further research finds that the improvement of internal control quality can weaken the promoting effect of financial independent directors’ compensation on stock price collapse to a certain extent, and the weakening effect is particularly evident in non-state-owned enterprises. The attendance frequency of financial independent directors cannot effectively suppress stock price collapse, but instead has a promoting effect.

## 1. Introduction

The 19th National Congress of the Communist Party of China pointed out that “we will deepen financial system reform, enhance the capability of financial services to support the real economy, improve the financial regulatory framework, and ensure that systemic risks are under control.” In recent years, there have been frequent incidents of stock market crashes, which not only damage the interests of investors and undermine their confidence but also spread through investor sentiment and other channels, triggering larger-scale stock market crashes that seriously affect the development of the real economy. Therefore, it is of great significance to explore the mechanisms and preventive measures of stock market crashes in order to improve the efficiency of the capital market and the quality of national economic development.

A stock market crash refers to a rapid and sharp decline in stock prices within a short period of time [1]. The occurrence of a stock market crash can be attributed to the self-interest motives of corporate management, who tend to conceal negative news in the long term and release all of it when the threshold of negative news is reached, causing a fatal impact on stock prices. To reduce the risk of stock market crashes, the most direct approach is to restrain the self-interest behavior of management and reduce the probability of hiding bad news. Supervising the behavior of management is the responsibility of independent directors in a company.

The independent director system is an important regulatory mechanism in modern corporate governance [2]. Its purpose is to introduce independent third parties into the board of directors who are not affiliated with the existing management team to supervise and reduce the control of internal personnel, in order to maintain fairness and protect the interests of small and medium-sized shareholders. In order to improve China’s corporate governance system, the government has forced the introduction of the independent director system. In 2001, the “Guiding Opinions on Establishing an Independent Director System in Listed Companies” (hereinafter referred to as the “Opinions”) was promulgated, which emphasizes that at least one-third of the members of the board of directors of listed companies should be independent directors, and at least one of them should have a financial or accounting background, known as the financial independent director. They rely on their professional knowledge to evaluate the information quality of listed companies, and are the “whistleblowers” of information quality. The “Opinions” also stipulate that listed companies should give independent directors appropriate allowances. The formulation of allowances is prepared by the board of directors, approved by the shareholders’ meeting, and disclosed in the annual report. Compared with the total compensation of executives, the compensation of independent directors is relatively small, but the incentive mechanism of interests will also promote the adverse impact of financial independent directors on the independence of enterprises [3, 4], exacerbating the tendency of violations of information disclosure regulations [5]. For a long time, there have been countless companies that have violated regulations and caused distortion of accounting information quality, and many companies have been exposed by the media with “explosive news”, causing their stock prices to plummet, resulting in independent directors being labeled as “figurehead independent directors”, “honorary directors”, “personal relationship directors”, and so on.

The independent director system is highly sought after by securities regulatory agencies around the world due to its ability to constrain opportunistic behavior by management. The direct manifestation of management’s opportunistic behavior is financial reporting, making the importance of independent financial directors self-evident. In theory, due to the minimal constraint that internal directors have on management, independent directors, constrained by their reputation mechanism, would implement more effective supervision [6]. However, empirical data shows that there are still debates about the governance function of independent directors [7], for example: factors such as reputation, experience, and part-time work can all affect the functional performance of independent directors [8, 9].

Different from previous studies, this article mainly explores the impact of financial independent directors’ compensation on stock price collapse from their perspective. Using data from listed companies from 2014 to 2020, the research found that: (1) there is a significant positive correlation between the compensation of financial independent directors and stock price collapse in listed companies. Higher compensation strengthens their interest relationship with management, reduces the independence and supervisory effect of independent directors. (2) From the perspective of property rights, the compensation of financial independent directors and stock price collapse are negatively correlated in state-owned enterprises, but not significant. In non-state-owned enterprises, there is a significant positive correlation between the two, indicating a higher probability of collusion or rent-sharing between financial independent directors and management in non-state-owned enterprises. (3) High-quality internal control can weaken the promotion effect of financial independent directors’ compensation on stock price collapse, which has a certain governance effect and is more pronounced in non-state-owned enterprises. (4) Further research found that the attendance of financial independent directors cannot effectively suppress stock price collapse, but instead has a promoting effect.

The contributions of this article are as follows: Firstly, existing literature mainly explores the stock price collapse from the perspectives of the geographic location, gender, and independent director system of independent directors, with few studies focusing on the financial compensation of independent directors. Secondly, current literature mainly examines compensation incentives from the perspectives of management or internal directors. Against the backdrop of significant differences in compensation for independent directors in China, this study helps to understand the impact of financial compensation for independent directors on stock price collapse. Finally, this article also has practical significance, as it can help companies avoid governance pitfalls (by increasing financial compensation for independent directors to suppress stock price collapse).

## 2. Literature review

### 2.1 Overview of independent director literature

In theory, independent directors are less likely to be influenced by internal management and have a higher degree of independence and motivation to supervise management due to their reputation. As the proportion of independent directors increases, corporate performance should also improve. However, actual research conclusions are inconsistent. Some scholars [7, 10, 11] have found that the higher the proportion of independent directors, the better the corporate performance. However, Li and Lai [12] and other scholars have found that there is no relationship between the proportion of independent directors and corporate performance.

Some literature considers the differences in independent director backgrounds and examines them from the perspective of individual characteristics. Yi and Xie [13] found that the network position of independent directors has a significant inhibitory effect on stock price collapses. Dong [14] analyzed the geographical location of independent directors and found that local financial independent directors can significantly reduce the risk of stock price collapse compared to non-local financial independent directors. Zheng et al. [15] found that independent directors with a media background, especially those who are older and have higher education, have an inhibitory effect on stock price collapses. Wu and Guan [16] found that celebrity independent directors have a certain degree of inhibitory effect on stock price collapses, and the inhibitory effect of female celebrity independent directors is more significant. Scholars have also found that hiring independent directors with banking backgrounds [8], accounting backgrounds [9, 17], and securities backgrounds [18] can not only improve information disclosure quality and optimize credit environment but also help improve corporate efficiency and performance. There are also different conclusions about the issue of independent director part-time jobs. Fich and Shivdasani [19] believe that hiring part-time independent directors can easily reduce corporate performance, but Ferris et al. [20] found that the number of part-time jobs does not affect the work input of independent directors, and companies with more part-time independent directors do not have more fraudulent or financial irregularities.

From the perspective of the reputation mechanism, many Western scholars [21] believe that the motivation for independent directors to perform their duties is market reputation, that is, to maintain their good reputation by reducing agency costs and resisting improper behavior of the company [22]. However, under the background of China’s economic transformation, the labor market for independent directors lacks effectiveness, and the reputation mechanism can only encourage them to avoid public violations through risk-avoidance behavior [23], but cannot motivate independent directors to perform their duties. The reputation mechanism of independent directors has not been effectively played in China [24].

Research on the compensation of independent directors can be roughly divided into two categories. One is the incentive hypothesis: giving independent directors allowances helps them play a supervisory role [25]. Because compensation incentives often account for more than 50% of independent directors’ total compensation [21], the higher the compensation incentive, the greater the responsibility and risk they bear, and the stronger their motivation to perform their duties, which can improve corporate governance efficiency and performance [25, 26]. The other is the collusion hypothesis: independent directors may abandon effective supervision of management in order to obtain high returns, resulting in “nepotism” behavior and loss of independence [27, 28]. The high or low compensation directly affects their expression of opinions, and the higher the compensation, the lower the probability of independent directors saying “no” [29]. Zhang et al. [30] believed that excessive or insufficient incentive compensation for independent directors can lead to different degrees of earnings management.

### 2.2 Literature review on stock price crash

Stock price crash, also known as Waterloo of stock price, is not only influenced by the macro market environment, but also by micro factors. Stock price crash is caused by the long-term concealment of bad news by the management team of a company, which is released when the bad news reaches a certain threshold, causing a fatal impact on the stock price, resulting in a stock price crash. This phenomenon not only damages the interests of small and medium shareholders, but also pushes the company to the brink of death. In existing research, the factors affecting stock price crash mainly focus on the following three aspects: first, many scholars analyze the relevant factors of agency conflicts and the correlation with stock price crash from the perspective of major shareholder shareholding ratio [31], The threat of non-controlling shareholders exiting [32], overinvestment [33], investor protection [34], relationship with online investors [35], excessive executive compensation [36], management overconfidence [37, 38], and management statements [39]; second, from the perspective of independent director-related characteristics, factors such as media background [15], celebrity independent directors [16], geographical location [14], and network location [13] are explored for their impact on stock price crash; third, some scholars analyze the impact of information asymmetry on stock price crash from the perspective of internal control [27] and financial report transparency [40].

In summary, research on the impact factors of independent directors and stock price crash is relatively abundant, but there is little research on the impact of financial independent directors’ compensation on stock price crash. The role of independent directors in the board of directors is to protect the interests of small and medium shareholders and reduce the improper behavior of the management team. The task of financial independent directors is to verify the information quality of financial reports and suppress the management team’s hoarding behavior (the management team tends to disclose good news and hide bad news for certain purposes) through regular disclosure, thereby suppressing stock price crash. The independent opinions expressed by financial independent directors directly affect the management team’s compensation income and promotion opportunities. Examining whether independent directors fulfill their duties from the perspective of financial independent directors’ compensation has certain research value and also provides theoretical basis for internal governance of enterprises.

## 3. Research hypothesis

### 3.1 Compensation of financial independent directors and stock price collapse

According to the current legal system, directors of a company, especially independent directors, not only participate in strategic decision-making but also supervise the company’s governance. In order to better perform their duties, they inevitably need to spend time and energy. Based on the rational economic person hypothesis, time and energy expenditure must be linked to compensation. Low compensation makes it difficult for independent directors to fulfill their duties [41]. Pound’s [42] study suggests that independent directors cannot do a job with low returns, high risks, and responsibility in the face of possible human capital market punishment, regulatory agency penalties, or investor lawsuits. Incentives for compensation have a certain impact on the performance of independent directors in their supervisory roles, such as the form of payment and the scale of compensation [17, 43]. Hope et al. [44] found that increasing compensation is the most direct incentive to improve the effectiveness of regulatory supervision. High compensation can motivate independent directors to be more diligent and responsible, solve the problem of low attendance rates [45], stimulate their willingness to actively supervise [26], increase the likelihood and number of dissenting behaviors [46, 47], prompt companies to replace CEOs when performance is poor [17], improve the efficiency of the board of directors’ supervision [48], increase the caution of independent directors on key issues [9], and stimulate their willingness to track board resolutions [26], inhibit corporate violations [49], and reduce the probability of concealing adverse information, thereby reducing stock price collapse.

In the fraud cases of large companies such as Enron, high compensation for independent directors strengthened their relationship with management, reduced the effectiveness of board supervision and the independence of independent directors [50], and led to the decline of corporate governance and business performance [28]. The “Opinions” does not specify the quantitative standards for independent director compensation, nor does it provide a clear response to the dispute over independent director compensation. This causes internal directors to consider not only cost but also their own private interests when selecting independent directors. They may choose to appoint financial independent directors who have a personal relationship with them, which makes independent directors more tolerant of management’s improper behavior [51] and more lenient towards management’s earnings management behavior [27], reduces regulatory efforts, and ultimately leads to a decline in corporate performance. At this time, the essence of compensation incentives is “collusion” with management. When compensation incentives exceed a certain standard, independent directors may choose to violate their fiduciary obligations, tacitly approve management’s improper behavior, and even engage in fraudulent activities with management, ignoring the interests of external stakeholders such as small and medium shareholders and institutional investors, making their supervisory role in name only, leaving hidden dangers for the eventual collapse of stock prices. The main task of financial independent directors is to supervise the accounting information of listed companies, restrain the “moral hazard” and “adverse selection” problems of management through corresponding supervision and evaluation systems, improve the quality of information disclosure, and protect the interests of small and medium shareholders. Independent director compensation is a key factor affecting the quality of financial reporting [27], and the independent opinions expressed by financial independent directors are directly related to the quality of information disclosure, which will ultimately affect the compensation and promotion of management. Compared with independent directors from other backgrounds, the motivation of management to collude with financial independent directors is more obvious, and the probability of bad news being hidden by the enterprise is higher, which increases the probability of stock price collapse.

Based on the above analysis, this article proposes research hypothesis 1:

H1a: Holding other conditions constant, the compensation incentives of financial independent directors have a significant inhibitory effect on stock price collapse.
H1b: Under certain conditions, the financial background of independent directors’ compensation incentives has a significant promoting effect on stock price collapse.

### 3.2 Scenario analysis of property rights

The relationship between independent director compensation incentives and accounting information quality may be related to corporate property rights. State-owned enterprises are regulated by departments such as the State-owned Assets Supervision and Administration Commission, and the selection criteria for independent directors are high [52] and appointed by the State-owned Assets Supervision and Administration Commission, so the “tricks” of internal directors can be effectively avoided. The internal and external governance structure of state-owned enterprises is relatively sound [53], the effectiveness of internal control quality is high, and the information production system is relatively complete. In addition, serving as an independent director of a state-owned enterprise can bring reputation, which is enough to promote independent directors to be more rigorous in key audit matters and information disclosure activities. Bad news that management tries to hide will be exposed, thereby reducing the probability of accumulating bad news. That is to say, independent director compensation incentives are not necessarily the key factor in stimulating the enthusiasm and diligence of independent directors in state-owned enterprises, but the reputation mechanism can better improve the quality of accounting information disclosure. In addition, state-owned enterprises are not only supervised by government and regulatory agencies [54], but also by public opinion [55]. At the same time, the high-level executive compensation of state-owned enterprises is mainly formulated by government departments with government attributes, and is subject to regulations such as the “Supplementary Provisions on Performance Evaluation of Leaders of Central Enterprises” and the “Guiding Opinions on Further Regulating the Management of Central Enterprise Leaders’ Compensation”. Therefore, the probability of state-owned enterprises arbitrarily increasing or decreasing independent director compensation is relatively low.

However, there are certain differences between non-state-owned enterprises and state-owned enterprises in terms of corporate governance and compensation formulation [56]. The information production system of non-state-owned enterprises is relatively imperfect, the effectiveness of internal control quality is weak, and there are deficiencies in internal and external governance mechanisms and organizational structures. The appointment of independent directors is also determined by the enterprise internally, and the state has not issued relevant regulations on the quantitative standards for independent director compensation, nor has it clearly rectified the market chaos of independent director compensation. In theory, with the complexity of the regulatory environment and the standardization of regulatory requirements, the compensation of independent directors in China should be linked to the risks they undertake. Enterprises should reward them by constantly increasing their compensation, so that high compensation can induce independent directors to undertake high risks and responsibilities, thereby improving the efficiency and quality of supervision, enhancing the quality of accounting information disclosure, and promoting stock price stability. However, empirical data shows that the reputation mechanism of independent directors has not been effectively played in non-state-owned enterprises in China [24], and compensation incentives are a direct means to improve regulatory effectiveness [44]. Financial independent directors may exhibit “nepotism” behavior in order to obtain high returns, which may lead to a loss of independence [27, 28], prompting management to adopt opportunistic behaviors such as intentionally hiding bad news and manipulating market value, resulting in information disclosed to the capital market by the company being “optimistic” information, and increasing the risk of stock price collapse.

Based on the above analysis, this article proposes research hypothesis 2:

H2: Under certain conditions, compared with state-owned enterprises, in non-state-owned enterprises, the higher the incentive for financial independent director compensation, the more promoting effect it has on stock price collapse.

## 4. Research design

### 4.1 Data source and sample selection

To avoid the impact of the global COVID-19 pandemic on the stock prices of companies from 2020 to 2022 and highlight the effectiveness of micro-level governance, this study selected listed companies from 2014 to 2020 as the initial research sample. To avoid the interference of abnormal data, the data was processed as follows: (1) financial and insurance companies were excluded; (2) samples with missing data were deleted; (3) ST-listed companies were removed; (4) samples with less than 30 weeks of annual weekly return data were deleted; (5) to eliminate outliers, Winsorize truncation was performed on continuous variables at the 1% and 99% percentiles. After the data processing, a total of 7, 551 sample observations were obtained. The data for this study mainly came from the Guotai database and Wind database, and the internal control index was obtained from the Dibo database.

### 4.2 Variable definition

#### 4.2.1 Stock price collapse risk

Drawing on the approach of Chen et al. [57] and Hutton et al. [40], the negative skewness coefficient (NCSKEW) and the ratio of upward and downward volatility of returns (DUVOL) were selected as the indicators for measuring the risk of stock price collapse. This study calculated the risk of stock price collapse using the following method.

First, the weekly data of stock i was used for regression in model (1) by year, and the individual weekly return rate W_i,t_was calculated based on the residuals.


$$r_{\mathrm{it}}=\alpha_{i}+\beta_{1}r_{M,t-2}+\beta_{2}r_{M,t-1}+\beta_{3}r_{M,t}+\beta_{2}r_{M,t+1}+\beta_{2}r_{{}_{M,t+2}}+\varepsilon_{i,\;t}$$ (1)

Among them, The cash dividend reinvestment yield for individual stock i in week t is represented by r_i,t_; r_M,t_ is the weighted average weekly capital yield of all stocks in the corresponding market based on their market capitalization in t weeks.; Excluding the impact of non-simultaneous transactions, add lagged and leading terms of the market return rate r_M_ to model (1). *ε*_i, *t*_ is the residual term. $W_{i,t}=Ln(1+\varepsilon_{i,t})$ represents the unique weekly return rate of a specific stock.

Secondly, the measure of stock price collapse risk is constructed based on W_i,t_.

(1) The negative skewness coefficient of returns, NCSKEW:

$${\mathrm{NCSKEW}}_{i,\;k}=-\frac{\left[n(n-{1)}^{3/2}\sum W^{3}{}_{i,\;k}\right]}{[(n-1)(n-2)(\sum W^{2}{}_{i,\;k}{)}^{3/2]}}$$ (2)

(2)The fluctuation ratio of returns, DUVOL.

$${\mathrm{DUVOL}}_{i,\;k}=\log\frac{\left[{(n}_{u}-1)\sum_{\mathrm{down}}W^{2}{}_{i,\;k}\right]}{\left[{(n}_{d}-1)\sum_{\mathrm{up}}W^{2}{}_{i,\;k}\right]}$$ (3)

In Model (2), n represents the number of trading weeks in a year for stock i. In Model (3), n_d_ and n_u_ respectively represent the number of weeks where the weekly return of stock i is higher or lower than the annual average return.

#### 4.2.2 Explanatory variables

Following the approach of Che, et al. [58] and Zheng, et al. [59], we measure the total annual compensation of independent directors in natural logarithm.

#### 4.2.3. Mediating variables

Drawing from the research of Lu, et al. [60] and Huang and Wu [61], we measure the quality of internal control by taking the natural logarithm of the DiBao China’s internal control index. This index comprehensively reflects the achievement of the five internal control objectives and the correction of internal control defects of listed companies in China.

#### 4.2.4. Control variables

Consistent with the studies of Gong [62], Zheng, et al. [59], and Wang, et al. [34], we control for the following factors: monthly average excess turnover rate (dturn), annual weekly stock return (ret), standard deviation of the company’s annual weekly return (sigma), leverage (lev), company size (size), growth (growth), the proportion of shares held by the largest shareholder (sds), board size (lns), and return on equity (ROE). The specific variable definitions are presented in Table 1.

**Table 1:** Variable description.

Type	Variable Name	Symbol	Variable Definition
Dependent variable	Negative skewness coefficient.	NCSKEW	Please refer to model (2) for specific calculations.
	Fluctuation ratio of income.	DUVOL	Please refer to model (3) for specific calculations.
Explanation of Variables	Compensation for Independent Directors of Finance	lnFSAL	Natural logarithm of the annual compensation of the financial independent director.
Control variables.	Monthly average excess turnover rate.	DTURN	Difference between the monthly turnover rate of stock i in year t and the monthly turnover rate in year t-1.
	Annual weekly return rate.	RET	Average weekly return rate of stock i in a specific week of year t.
	Annual weekly rate of return standard deviation	SIGMA	Annual standard deviation of the specific weekly return rate of stock i in year t.
	Asset-liability ratio	LEV	Total liabilities/total output value.
	Company size	SIZE	Natural logarithm of the total assets of the company.
	Growth potential	GROWTH	Growth rate of the main business income.
	Percentage of shares held by the largest shareholder	SDS	Percentage of shares held by the largest shareholder.
	Board size	lnS	Natural logarithm of the number of directors on the board of the company.
	Net asset net profit margin	ROE	Net profit/total shareholder equity.
Mediating variable.	Internal control index	LnIQ	Natural logarithm of the “internal control index” disclosed in the Di Bo database.

### 4.3 Model design

Based on hypothesis H1, a regression model (4) is established, where Crash_it_ is the stock price crash variable, and NCSKEW and DUVOL are respectively the skewness and downside volatility of company i in year t.


$${\mathrm{Crash}}_{\mathrm{it}}=\beta_{0}+\beta_{1}{\mathrm{lnFSAL}}_{i,t‐1}+\beta_{2}{\mathrm{Controls}}_{i,t‐1}+\sum Year+\sum ind+\varepsilon_{i,t-1}$$ (4)

### 4.4 Empirical analysis

#### 4.4.1. Descriptive statistics

After the data collection and processing, the descriptive statistics of the main variables are shown in Table 2. The mean values of NCSKEW and DUVOL are -0.375 and -0.233, respectively, and the difference between the two is not significant, but overall they are negative. From the range between the maximum and minimum values, there is a large difference in the risk of stock price collapse among companies, which is consistent with previous studies. The average salary of financial independent directors is 10.87, with a maximum of 12.9 and a minimum of 0, indicating a significant difference in the incentive intensity of financial independent directors among different listed companies, with a difference of nearly 13 times between the highest and lowest salaries. The average borrowing of sample companies accounts for 46.1% of the total assets, indicating that listed companies are using financial leverage to some extent. The average income growth rate of sample companies is stable at around 45%. The average shareholding ratio of major shareholders is 1/3 of all shares. At the same time, the mean value of the net asset profit margin (i.e. sustainable growth rate) of the companies is 0.93%.

**Table 2:** Descriptive statistical analysis of main variables.

Variable	N	mean	sd	p50	max	min
NCSKEW	7551	-0.357	0.788	-0.330	4.311	-5.112
DUVOL	7551	-0.233	0.710	-0.257	3.681	-3.202
lnFSAL	7551	10.90	2.300	11.29	14.00	0
DTURN	7551	46.49	36.85	35.65	356.0	1.385
RET	7551	-0.0020	0.00260	-0.00160	0.00240	-0.00550
SIGMA	7551	0.0004	0.995	-0.0789	10.32	-7.789
LEV	7551	0.461	0.200	0.459	0.996	0.00910
SIZE	7551	22.59	1.306	22.42	28.22	16.00
GROWTH	7551	0.450	7.099	0.0796	431.2	-1
SDS	7551	33.40	14.63	31.28	89.99	3.620
lnS	7551	2.135	0.196	2.197	2.890	1.099
ROE	7551	0.0093	1.089	0.0634	2.324	-50.08

#### 4.4.2. Regression analysis

Assuming that the regression results of H1 are shown in Table 3 (Regression 1). The results indicate that the lnFSAL of financial independent directors’ compensation is significantly positively correlated with the stock price collapse indicator Cash at the 5% level. This suggests that the compensation incentive for financial independent directors not only fails to curb the stock price collapse but also promotes it. That is, the collusion or rent-sharing event between financial independent directors, internal directors, and management is a high-probability event. If the compensation incentive for financial independent directors is increased, the transparency of financial information of listed companies will not only decrease but also the fraudulent behavior of management will become more rampant, resulting in an increase in the frequency of stock price collapses and ultimately harming the interests of small and medium-sized shareholders. This also provides support for hypothesis H1b.

**Table 3:** Regression analysis of main variables.

Variable	Return 1		Return to 2. State-owned enterprise group.		Return 2. Non-state-owned Group.	
	(1)	(2)	(3)	(4)	(5)	(6)
	NCSKEW	duvol	NCSKEW	duvol	NCSKEW	duvol
lnFSAL	0.0097**	0.0079**	-0.0004	-0.0022	0.0151**	0.0135**
	(0.039)	(0.047)	(0.958)	(0.715)	(0.007)	(0.005)
DTURN	-0.0037***	-0.0048***	-0.0055***	-0.0082***	-0.0031***	-0.0038***
	(0.000)	(0.000)	(0.000)	(0.000)	(0.000)	(0.000)
RET	57.8422***	27.8943**	47.1076*	-22.5536	64.0612**	63.0109***
	(0.000)	(0.028)	(0.056)	(0.300)	(0.001)	(0.000)
SIGMA	0.0214**	-0.0240**	0.0040	-0.0368**	0.0259**	-0.0214*
	(0.029)	(0.005)	(0.816)	(0.014)	(0.042)	(0.051)
LEV	0.1265	0.0571	0.5459**	-0.0337	-0.0088	0.1375
	(0.290)	(0.564)	(0.016)	(0.866)	(0.951)	(0.218)
SIZE	0.0457*	0.0299	0.0253	0.0455	0.0709**	0.0377
	(0.072)	(0.155)	(0.603)	(0.289)	(0.026)	(0.130)
GROWTH	-0.0009	-0.0006	-0.0040	-0.0030	0.0004	0.0001
	(0.604)	(0.747)	(0.209)	(0.277)	(0.749)	(0.955)
SDS	-0.0040**	-0.0029*	-0.0099**	-0.0089**	-0.0012	-0.0025
	(0.040)	(0.066)	(0.002)	(0.002)	(0.639)	(0.207)
lnS	-0.1053	0.0168	-0.2971*	-0.2247	-0.0099	0.1914
	(0.318)	(0.867)	(0.083)	(0.138)	(0.944)	(0.113)
ROEA	-0.0065	0.0027	-0.0381	-0.0610**	-0.0066	0.0068
	(0.620)	(0.709)	(0.196)	(0.019)	(0.727)	(0.385)
Constant	-0.9195	-0.6350	0.0220	-0.1916	-1.7302**	-1.1777**
	(0.103)	(0.190)	(0.984)	(0.841)	(0.016)	(0.034)
Industry/year	Control	Control	Control	Control	Control	Control
N	7551	7551	2679	2679	4872	4872

* p<0.1

** p<0.05

*** p<0.001

Assuming that the regression results of H2 are shown in Table 3 (Regression 2 for state-owned enterprises and Regression 2 for non-state-owned enterprises). From the results of Regression 2 for state-owned enterprises, it can be seen that the compensation of financial independent directors is negatively correlated with the stock price collapse (Cash) in state-owned enterprises, but not significantly. This indicates that the compensation incentive for financial independent directors has a certain inhibitory effect on the stock price collapse in state-owned enterprises, but the effect is not significant. This may be related to the special nature of state-owned enterprises and relevant legal provisions, that is, the willingness of internal management to obtain private benefits from the level of independent directors is suppressed. In order to maintain their reputation and promotion opportunities, financial independent directors will continue to maintain their independence and objectivity, actively improve the transparency and quality of accounting information, and thus curb the fraudulent and illegal behavior of management.

From the regression results of Regression 3 for non-state-owned enterprises, it can be seen that the stock price collapse Cash is significantly positively correlated with the compensation of financial independent directors at the 5% level in non-state-owned enterprises. This indicates that the compensation incentive for financial independent directors has a reverse effect in non-state-owned enterprises, that is, high compensation prompts financial independent directors to be less objective in performing their supervisory functions. This may be related to the lack of relevant regulations on compensation in our country, leading to an unfavorable situation in the management of independent director compensation in non-state-owned enterprises. It also confirms the public’s saying of “figurehead independent directors” and “personal favor independent directors”, that financial independent directors may help management hide bad news or even collude in fraudulent activities and fail to perform their supervisory functions. Furthermore, it indicates that the quality and transparency of accounting information in non-state-owned enterprises are poor, and small and medium-sized investors need to use non-state-owned enterprises’ disclosure information with caution when making investment decisions. This also provides support for hypothesis H2.

### 4.5. Robustness testing

To ensure the robustness and reliability of the testing results, this paper employs the following three modes of testing:

#### 4.5.1. Multicollinearity and heteroscedasticity testing

To test the robustness of the model, the multicollinearity and heteroscedasticity of the model (4) are tested. The VIF values in the multicollinearity regression process are far less than 10, all within an acceptable range, indicating that there is no serious multicollinearity. The P value for heteroscedasticity is 39.36%, indicating that there is also no heteroscedasticity.

#### 4.5.2. Changing the method of measuring stock price crash risk

Drawing on the research of Zhang et al. [63], there is a strong positive correlation between stock price crashes and stock price synchronicity. The higher the stock price synchronicity, the more serious the phenomenon of “rising and falling together,” and the lower the company information content contained in stock price fluctuations. This increases the risk of hidden bad news and thus the risk of stock price crashes.

First, following the approach of Durnev et al. [64] and Jin and Myers [65], regression estimation is performed using Eq (5) to obtain the goodness of fit R^2^ of model (5) as a measure of stock price synchronicity. Since the range of values for the goodness of fit is 0–1, which does not conform to a normal distribution, Eq (6) is used for adjustment.


$$R_{\mathrm{i,t}}{=\beta }_{0}{+\beta }_{1}{*R}_{\mathrm{m,t}}{+\beta }_{2}{*R}_{\mathrm{I,t}}+\varepsilon$$ (5)

$$\mathrm{Synch}=\ln\frac{R^{2}}{{1‐R}^{2}}$$ (6)

The regression results, as shown in Table 4 (regression 3 column 7), indicate that the compensation of financial independent directors is also significant at the 10% level with stock price collapse, supporting the original hypothesis.

**Table 4:** Robust regression analysis of the main variables.

Variable	Regression3 measuresthe synchronicity of stock prices in the event of a stock market crash.	Regression 4 adjusts the predicted range of stock market crash risk.		Regression 5 2SLS	
	(7)	(8)	(9)	(10)	(11)
	synch	NCSKEW	DUVOL	NCSKEW	DUVOL
lnFSAL	0.0092*	0.0096**	0.0073*	0.0780	0.1887
	(0.097)	(0.040)	(0.066)	(0.082)*	(0.000)***
NCSKEWt-1		-0.0681			
		(0.405)			
DUVOLt-1			-0.1535***		
			(0.000)		
Control variables	Control	Control	Control	Control	Control
Industry/year	Control	Control	Control	Control	Control
N	7551	7551	7551	7551	7551

* p<0.1

** p<0.05

*** p<0.001

#### 4.5.3. Changing the prediction interval of stock price collapse risk

Drawing on Su’s [66] research, in order to avoid the lag of stock price collapse, we added the leading period Cash to Model (4). The results showed that the compensation of financial independent directors and stock price collapse are still significant at the 10% level, supporting the above hypothesis.

#### 4.5.4. Instrumental variable analysis

In order to avoid endogeneity issues in the model, this section uses a two-stage least squares method (2SLS) to address potential endogeneity problems in the model. This study selects the lagged period of the explanatory variable lnFSAL as the instrumental variable, resulting in the estimation results of the two-stage least squares method shown in Table 4 Regression 5. The compensation of independent directors in relation to stock market crashes remains significant at the 10% level, supporting the original hypothesis and indicating that the model is robust enough.

## 5. Further analysis

### 5.1. Study on governance path

Internal control is a risk management mechanism that helps alleviate conflicts of interest at various levels. On one hand, it has an impact on the quality and reliability of the corporate information production system [67]. Research has found that the more effective internal control is, the higher the quality of internal control, and the higher the level of information quality within a company [68]. It also reduces the probability of significant misreporting in the company’s performance reports and minimizes reporting errors [69]. On the other hand, high-quality internal control can improve communication efficiency to a certain extent, effectively balance power relationships between departments, and restrain management from manipulating information and engaging in self-interest behaviors, thus reducing agency costs [61]. Enhancing the effectiveness of independent directors is an important component of internal control management. High-quality internal control not only creates a transparent environment for each functional department to perform its duties but also improves the effectiveness of supervision.

The above research confirms that there is a positive relationship between the compensation of independent directors and stock price collapse. In order to improve this situation, this article uses the mediation effect model to test whether the improvement of internal control quality can weaken the positive relationship between the compensation of independent directors and stock price collapse, and play a role in suppressing stock price collapse. The model is mainly divided into three steps. Firstly, estimate the baseline regression (4) to test whether the compensation of independent directors significantly affects stock price collapse. Secondly, replace the dependent variable with the internal control index lnIQ to obtain model (7). If the coefficient of *α*_1_ is significantly negative, it indicates that the improvement of internal control quality can suppress the increase in compensation of independent directors. Finally, add the internal control index lnIQ to model (4) to obtain model (8). If only the coefficients of *α*_1_ in model (7) and *θ*_2_ in model (8) are significantly negative, it indicates that internal control quality is a significant mediating variable. If the coefficient of $\theta_{1}^{'}$ in model (8) is also negative, it indicates that this mediation effect is a partial mediation effect.


$${\mathrm{lnIQ}}_{\mathrm{it}}=\alpha_{0}+\alpha_{1}{\mathrm{lnFSAL}}_{\mathrm{i,t}‐1}+\alpha_{2}{\mathrm{Controls}}_{\mathrm{i,t}‐1}+\sum Year+\sum ind+\varepsilon_{i,t-1}$$ (7)

$${\mathrm{Crash}}_{\mathrm{it}}=\theta_{0}+\theta_{1}{\mathrm{lnFSAL}}_{\mathrm{i,t}‐1}+\theta_{2}{\mathrm{lnIQ}}_{\mathrm{i,t}}+\theta_{3}{\mathrm{Controls}}_{\mathrm{i,t}‐1}+\sum Year+\sum ind+\varepsilon_{i,t-1}$$ (8)

In models (7) and (8), lnIQ_it_ represents the internal control quality index of enterprise i in year t, and the definitions of other variables are consistent with model (4). The regression results of the mediating effect are shown in Table 5. Regressions 6, 7, and 8are the results of models (4), (7), and (8), respectively.From regression 7, it can be seen that the correlation coefficient between lnIQ and lnFSAL is negative and significant at the 10% level. Regression (8) shows the coefficient of $\theta_{1}^{'}$ is significant positive correlation at the 10% level, still supporting hypothesis H1b, and the coefficient of *θ*_2_ is significant negative correlation at the 5% level, indicating that internal control quality only plays a partial mediating role. This suggests that the positive correlation between CEO compensation and stock price crash can be weakened by improving internal control quality. From the analysis of property rights, as shown in regressions 9 and 10, the relationship between Crash and lnFSAL is consistent with the verification results of hypothesis 2, and the inhibitory effect of internal control quality is more significant in non-state-owned enterprises. Therefore, improving the internal control quality of non-state-owned enterprises has a significant inhibitory effect on stock price crashes.

**Table 5:** Regression analysis of the mediation effects.

	regression 6		regression 7	regression 8		regression 9		regression 10	
	(12)		(13)	(14)		(15) State-owned enterprise Group.		(16) Non-state-owned Group.	
	NCSKEW	DUVOL	lnIQ	NCSKEW	DUVOL	NCSKEW	DUVOL	NCSKEW	DUVOL
lnFSAL	0.0097**	0.0079**	-0.0015*	0.0116**	0.0075**	-0.0018	-0.0022	0.0145**	0.0129**
	(0.039)	(0.047)	(0.093)	(0.011)	(0.049)	(0.798)	(0.722)	(0.010)	(0.008)
lnIQ				-0.2519***	-0.2491***	-0.2033*	-0.1498	-0.2782***	-0.3111***
				(0.000)	(0.000)	(0.076)	(0.132)	(0.001)	(0.000)
Control variables			Control	Control	Control	Control	Control	Control	Control
Industry/year			Control	Control	Control	Control	Control	Control	Control
N			7551	7551	7551	2679	2679	4872	4872

* p<0.1

** p<0.05

*** p<0.001

### 5.2 Examining whether the attendance of financial independent directors suppresses stock price collapse

The direct pathway for independent directors to fulfill their duties is through attending board meetings, and the frequency of their attendance can reflect the extent of their efforts to a certain degree. The higher the attendance rate of independent directors, the more it can restrain the expropriation behaviors of controlling shareholders [70]. Furthermore, it can also improve company performance [71] and company value [72]. In order to investigate the relationship between the compensation and attendance of financial independent directors and stock price collapse, we constructed models (9) and (10), which replaced the dependent and independent variables based on model (4). Lnattende represents the natural logarithm of the average number of board meetings attended by financial independent directors in a year. The results, as shown in Table 6, indicate a significant positive correlation between the compensation and attendance of financial independent directors at the 1% level, suggesting that compensation is positively related to the level of effort of independent directors, which is consistent with previous research. The attendance of board meetings is positively correlated with stock price collapse at the 10% level, indicating that increasing the attendance of financial independent directors cannot effectively suppress but instead promotes stock price collapse. Moreover, there is a significant positive correlation between the two in non-state-owned enterprises, meaning that the more meetings attended by financial independent directors in non-state-owned enterprises, the higher the probability of collusion between independent directors and management to share rent, thereby accelerating stock price collapse.

**Table 6:** Financial independent directors’ attendance at board meetings and stock price collapse.

	regression 11	regression 12		regression 13		regression 14	
				(2) Non-state-owned Group.		(3)State-owned enterprise group	
	lnattende	NCSKEW	duvol	NCSKEW	duvol	NCSKEW	duvol
lnattende		0.0588***	0.0286*	0.0907***	0.0367**	0.0346	0.0105
		(0.001)	(0.057)	(0.000)	(0.048)	(0.265)	(0.698)
lnFSAL	0.1136***						
	(0.000)						
Control variables		Control	Control	Control	Control	Control	Control
Industry/year		Control	Control	Control	Control	Control	Control
N		7551	7551	4872	4872	2679	2679

* p<0.1

** p<0.05

*** p<0.001

## 6. Research conclusion and implications

This paper explores whether the compensation of financial independent directors promotes or inhibits stock price collapse by focusing on the compensation of financial independent directors of listed companies from 2014 to 2020. The research results show that there is a significant positive correlation between the compensation of financial independent directors and stock price collapse in listed companies. That is, higher compensation for financial independent directors strengthens their relationship with the management, reduces the independence and supervisory effectiveness of independent directors. From the analysis of the property rights of Chinese enterprises, the compensation of financial independent directors in state-owned enterprises is negatively correlated with stock price collapse, but not significantly. In non-state-owned enterprises, there is a significant positive correlation between the two, indicating that the probability of collusion or rent-sharing between financial independent directors and management is higher in non-state-owned enterprises. In response to the positive correlation between the compensation of financial independent directors and stock price collapse, this paper explores ways to weaken the relationship between the two. The results show that high-quality internal control can weaken the relationship to some extent, and has a significant inhibitory effect in non-state-owned enterprises. Further research shows that the attendance of financial independent directors cannot effectively suppress stock price collapse, but instead has a promoting effect.

Agency conflicts have always been a difficult problem in corporate governance. As an effective mechanism to solve this conflict, the independent director system has been highly valued by securities regulatory agencies in various countries. China also introduced this system in 2001, but the role of independent directors has always been controversial. Compared with independent directors of other backgrounds, financial independent directors, as the gatekeepers of financial reports, have higher requirements for independence and objectivity. To effectively perform the functions of financial independent directors, it is necessary to spend a lot of time and energy on gatekeeping. Based on the economic man hypothesis, financial independent directors will certainly require an increase in compensation, but this may also lead to collusion between financial independent directors and internal management. To effectively perform the functions of financial independent directors, companies need to evaluate the quality of their internal control. If the effectiveness of internal control is low, increasing the compensation of financial independent directors will not only reduce the quality of information disclosure but also increase the probability of stock price collapse. On the contrary, financial independent directors can actively perform their duties and play an effective supervisory role. At the same time, we call on the government to introduce relevant regulations on the compensation of non-state-owned enterprise independent directors, and establish corresponding benchmarks for non-state-owned enterprises in setting independent director compensation.

This article has certain limitations. Taking a macro perspective and using International Financial Reporting Standards (IFRS) to prepare financial statements can improve comparability and reliability of financial statements, reduce information costs and risks, and help boost investor market confidence [73, 74]. This is also one of the reasons why China’s accounting standards are gradually converging with international standards. Further exploration is needed to analyze the impact of adopting IFRS on stock market crashes in China.

## Supporting information

S1 Data (XLSX)
